# Vincristine-Induced Bilateral Vocal Cord Palsy in an Adult Male With Diffuse Large B-Cell Lymphoma

**DOI:** 10.7759/cureus.18591

**Published:** 2021-10-08

**Authors:** Samuel Leedman, Stephanie Flukes

**Affiliations:** 1 Department of Otolaryngology, Head and Neck Surgery, Fiona Stanley Hospital, Perth, AUS; 2 Division of Surgery, University of Western Australia, Perth, AUS

**Keywords:** airway, complication, lymphoma, vincristine, vocal cord palsy

## Abstract

Acute bilateral vocal cord palsy (BLVCP) is an airway emergency. Elucidating the underlying cause is imperative to enable appropriate management. Vincristine-related neurotoxicity is a potentially reversible cause of BLVCP and is rarely described in the literature. We report a case of a 65-year-old man who presented with acutely worsening dyspnoea and stridor following his fifth cycle of rituximab, cyclophosphamide, doxorubicin, vincristine, and prednisolone (R-CHOP) chemotherapy for hematological malignancy. His airway limitation was managed with supportive measures until he was able to compensate, at which point he was discharged home. His chemotherapy regimen was altered and he underwent serial examinations until he regained full vocal cord mobility at three months following his initial presentation. Through reporting this case, we hope to raise awareness of the potential for vincristine to cause sudden BLVCP and resultant airway deterioration, as well as emphasize the reversible nature of the condition with prompt cessation of therapy.

## Introduction

Acute bilateral vocal cord paralysis (BLVCP) is an airway emergency. There is a range of potential causes, including trauma to the neck or chest (surgical or accidental), compressive lesions along the course of the recurrent laryngeal nerves (including cervical or mediastinal lymphadenopathy as well as thyroid disease), or mononeuropathies, which can develop due to autoimmune, infectious, or toxic causes [1]. Workup involves structural imaging to exclude compressive lesions, as well as biochemistry to exclude autoimmune causes. In the event that these investigations are normal, toxic mononeuropathies are considered as a diagnosis of exclusion. We report a rare case of vincristine-induced BLVCP to raise awareness of this uncommon etiology. The patient provided written consent to be included in this report.

## Case presentation

**Figure 1:**
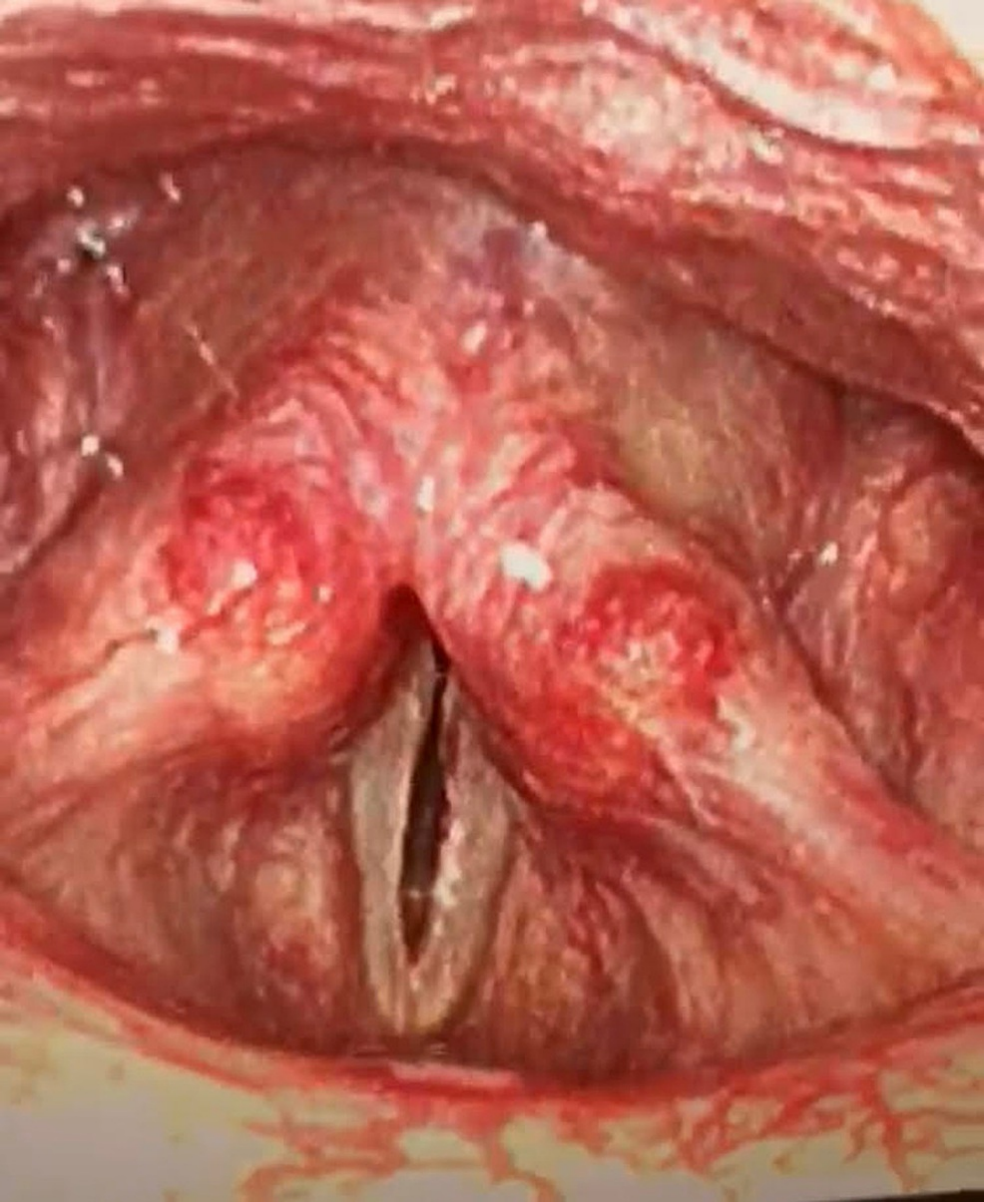
Clinical image of flexible laryngoscopic view showing bilateral vocal cords fixed in the paramedian position.

A 65-year-old man presented to the emergency room with a 10-day history of worsening dyspnea. His medical history was significant for active diffuse B-cell lymphoma (DLBCL), and he had recently completed his fifth cycle of rituximab, cyclophosphamide, doxorubicin, vincristine, and prednisolone (R-CHOP) chemotherapy 12 days prior to hospital presentation. On examination, he was in obvious respiratory distress. He was tachypneic and tachycardic but maintaining his oxygen saturation. He had audible biphasic stridor and was able to speak in single words only. Transnasal flexible laryngoscopy revealed that the vocal cords were structurally normal but fixed in the paramedian position (Figure 1).

The remainder of the head and neck examination was unremarkable. He had previously undergone transnasal flexible laryngoscopy for an unrelated otolaryngology presentation and was found to have normal vocal cord movement at that time, which lead to the conclusion that the BLVCP was a new development. There had been no preceding laryngeal trauma or intubation in the interim.

The patient was admitted for further workup and management. Intravenous dexamethasone and nebulized epinephrine were administered with minimal effect. The patient gradually compensated over the next 24 hours and a tracheostomy was avoided. He denied dysphagia and continued on a normal oral diet. A computed tomography scan of the head, neck, and chest showed no structural lesions along the course of the recurrent laryngeal nerves to account for the acute BLVCP, and laboratory studies showed no evidence of infectious or autoimmune cause for his presentation. In the absence of other possible etiologies, it was presumed that toxicity related to the R-CHOP regimen was the cause of the BLVCP. The patient was discharged home after three days of observation with a clinical improvement in his respiratory effort; however, he continued to have complete BLVCP on serial flexible laryngoscopy. Further chemotherapy doses were canceled pending resolution of the acute BLVCP.

The patient was reviewed four weeks post-discharge, at which point repeat flexible laryngoscopy showed a flicker of movement in both vocal cords. Repeat flexible laryngoscopy at three months showed near-complete recovery of vocal cord movement bilaterally. He was recommenced on an alternative chemotherapy regimen with a good response and remains disease-free with normal laryngeal function at three years following his initial presentation.

## Discussion

Identification of the underlying cause of spontaneous acute BLVCP is important to enable the correction of reversible causes and to prognosticate recovery. In the case described, the R-CHOP chemotherapy regimen was identified as the likely etiology. Of the five component medications that make up the R-CHOP regimen, vincristine was considered the most likely cause of the BLVCP. Vincristine is a vinca alkaloid chemotherapy drug that works by interfering with microtubule organization during mitosis. This mechanism of action leads to the unwanted side effect of vesicle-mediated transport within nerve axons and consequent axonopathy [2].

Vincristine-related neurotoxicity is well described, although it most commonly affects the peripheral motor and sensory nerves [2]. Involvement of the cranial nerves is less common. BLVCP is infrequently described in the literature, with fewer than 20 cases reported [3-6]. Of these, the majority occurred in pediatric patients. Treatment involves immediate cessation of vincristine, with the management of the airway driven by clinical need. Recovery time to normal vocal cord mobility is reported to range from two weeks to nine months (most commonly three to six months). One series reported re-treatment with a lower dose of vincristine at a later date [6].

The majority of previously reported cases have appeared in hematology/oncology and clinical pharmacology journals. We report this case in order to raise awareness of the condition from an otolaryngology perspective. Management of the airway is an important consideration in these patients. Our case highlights that some patients are able to compensate for their BLVCP. In these cases, close observation can be appropriate. However, the clinical course of BLVCP is uncertain, and prompt placement of tracheostomy must always be at the forefront of any plan when dealing with these patients.

## Conclusions

BLVCP is a rare but potentially life-threatening side effect of vincristine chemotherapy. The condition is reversible upon cessation of vincristine and so hematologists and otolaryngologists should be aware of this uncommon cause of BLVCP.
